# Bilateral Pediatric Myelin Oligodendrocyte Glycoprotein Antibody-Associated Disease (MOGAD) Optic Neuritis: A Case Report

**DOI:** 10.7759/cureus.74883

**Published:** 2024-11-30

**Authors:** Nadhirah Ahmad Fauzi, Abd Bari Muhd-Syafi, Abdul Hadi Rosli, Wan Muhammad Najib Wan Mahmud Sabri, Aidila Jesmin Jabbari

**Affiliations:** 1 Ophthalmology, International Islamic University Malaysia, Kuantan, MYS; 2 Paediatrics, Sultan Ahmad Shah Medical Centre, International Islamic University Malaysia, Kuantan, MYS

**Keywords:** mogad, mri, multiple sclerosis, nmosd, optic neuritis

## Abstract

Optic neuritis (ON) is defined as an acquired disorder of the optic nerve that may be associated with demyelinating diseases or infectious or inflammatory processes. In children, the manifestation of this condition differs from that in adults, where it typically presents with bilateral papillitis subsequent to a preceding viral illness. Nonetheless, the main concern for practitioners is the possibility of its conversion to multiple sclerosis (MS). In recent years, there has been increased awareness regarding differentiating MS from other demyelinating ON phenotypes, namely, neuromyelitis optica spectrum disorder (NMOSD) and myelin oligodendrocyte glycoprotein antibody-associated disease (MOGAD). Despite the clinical similarities among these three entities, they vary in terms of pathophysiology, clinical course, treatment approaches, and prognostication. In this paper, we highlight the case of a child with MOGAD ON who was clinically severe at presentation and has typical features of ON and the appropriate investigation and treatment done to achieve complete visual recovery.

## Introduction

Optic neuritis (ON) is a neuro-ophthalmic emergency. It is a rare but important cause of unilateral or bilateral acute-onset visual loss in children [1-3]. However, they tend to have good visual recovery despite initially presenting with more profound visual loss than adults [2]. Secondary causes of ON are more common in children; thus, it is important to investigate the presence of associated disease states. In recent years, there has been increased awareness to differentiate the different ON phenotypes, namely, multiple sclerosis (MS), neuromyelitis optica spectrum disorder (NMOSD), and myelin oligodendrocyte glycoprotein antibody-associated disease (MOGAD). Anti-MOG and anti-aquaporin-4 (AQP4) antibodies are laboratory investigations that are mainly used in detecting this difference in ON phenotypes. Apart from these laboratory investigations, a comprehensive examination is vital in the management of pediatric ON, and this includes history taking, optic nerve examination, imaging, and serology testing [3-4]. 

## Case presentation

An eight-year-old girl presented to the emergency department with bilateral eye acute visual loss for one day. The child complained to her parents that she was unable to perform her school homework because she could not see her books properly. The parents are also unable to get the child to focus on the objects in front of her. The visual loss was associated with pain during eye movement. The parents reported she had a brief episode of viral fever that happened around two weeks before the start of the visual complaint. The viral fever was self-limiting and did not require a visit to health facilities. She also experienced intermittent severe headaches for a duration of two weeks. The child was less active during the period of headache. The headache was relieved with rest. Otherwise, there was no history of red eyes or trauma to the eyes. There was no recent travel, swimming in a river or pool, or pets at home. There was no history of seizures to indicate any associated neurological symptom

**Figure 1:**
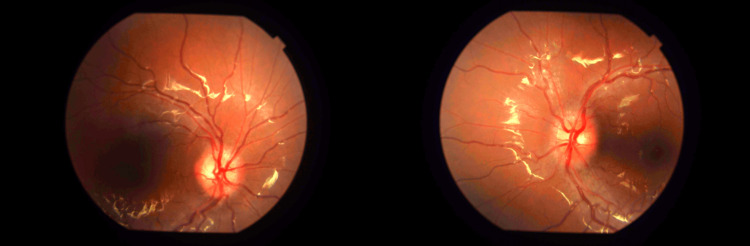
Right and left fundus photographs of the eye at the day of presentation with the optic disc in central position. There is bilateral disc swelling with loss of clear margin

On examination, the right eye's visual acuity was 1/120, and on the left eye, there was perception of light. A positive relative afferent pupillary defect (RAPD) was noted on the left eye. There was pain on eye movement in general and not associated with any specific gaze direction. Pupil examination revealed a mid-dilated pupil on the left that was not responsive to light. No lid swelling, ptosis, extraocular movement limitation, conjunctival injection, or corneal pathology noted. Fundoscopy examination demonstrated bilateral optic disc swelling (Figure 1). There were no signs of posterior uveitis such as vitritis, retinitis, vasculitis, or choroiditis. The macula was flat with no presence of macula star or exudate. We were unable to perform or document other optic nerve function tests, such as color vision, visual field testing, and contrast sensitivity because the patient presented with marked visual impairment. The rest of the neurological examination, including cerebellar signs, was normal. 

Initial blood work was negative for infection and inflammation, with normal values of white blood cell count at 10 x 10^9^/L (normal value: 4-11 x 10^9^/L), erythrocyte sedimentation rate at <29mmol/hour, C-reactive protein of <0.5 mg/dL, and negative serologies (Cytomegalovirus (CMV), Herpes simplex virus (HSV), Rubella, and Toxoplasma). Urinalysis ruled out urinary tract infection. 

**Figure 2:**
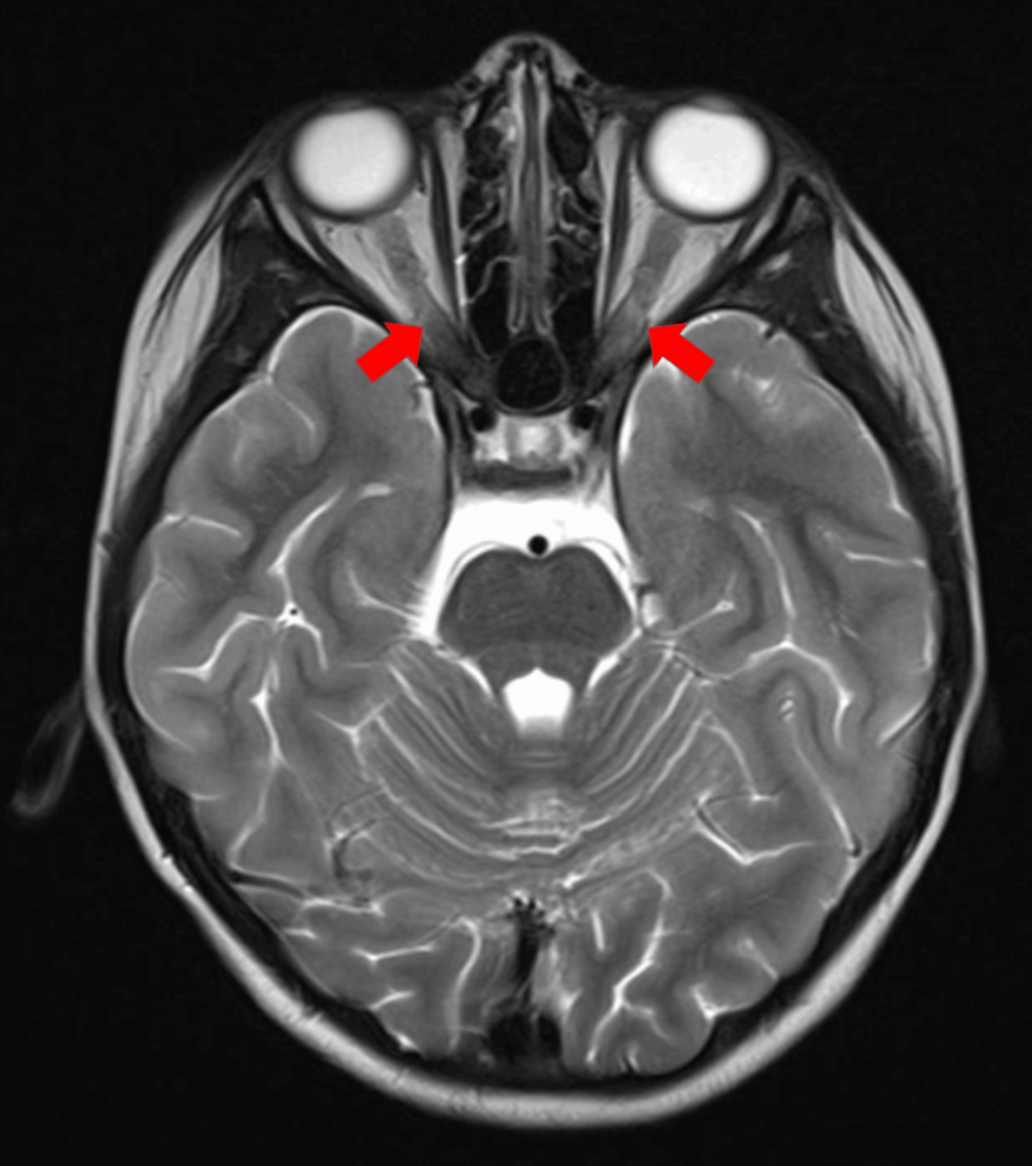
MRI brain and orbit T2-weighted post gadolinium showing symmetrically thickened optic nerve till the optic chiasm (red arrow)

Magnetic resonance imaging (MRI) was performed the next morning and revealed the presence of bilateral ON with tiny foci of hyperintense signal intensity in bilateral white matter (Figure 2). Cerebrospinal fluid (CSF) analysis revealed a normal composition, with a normal lumbar puncture opening pressure. CSF culture also came back negative for any growth. 

Intravenous methylprednisolone therapy of 30 mg/kg/day in three divided doses for five days were initiated then continued with oral prednisolone 1 mg/kg/day. The oral prednisolone was tapered down over the period of three months. At day 10 of illness, patient vision improved to a visual acuity of 6/12 on the right eye and 2/120 on the left eye. 

An internationally standardized cell-based assay (CBA) for antibodies later came back as positive anti-MOG and a negative anti-AQP4. A diagnosis of MOG-associated demyelinating ON was made. 

A follow-up MRI of the brain and orbit was done at six months after the first presentation. It shows evidence of resolving bilateral ON with similar appearance of white matter lesion. The patient was also scheduled and underwent MRI of the spine as a routine workout to rule out transverse myelitis. The result of the MRI was not suggestive of transverse myelitis as there were no presence of white matter lesion on the MRI. At the sixth-month follow-up, the patient showed full recovery with final visual acuity of 6/6 and intact optic nerve function test in both eyes. The child was planned for a follow-up at six-monthly intervals for the first two years upon completion of visual recovery.

## Discussion

Pediatric ON has not been extensively studied due to its rarity. The common presentation of pediatric ON is bilateral papillitis with marked visual impairment following a viral illness [1-3]. However, despite poor visual acuity at presentation, the visual prognosis is good with complete visual recovery. When dealing with a case of ON, the question that should be addressed is the risk of conversion to MS [5]. Of late, there is increased awareness in differentiating the phenotypes of demyelinating ON, namely, NMOSD and MOGAD [6]. 

Positive anti-AQP4 antibodies confer that a patient is at high risk for NMOSD. In MOGAD, MOG antibodies are positive whereas having negative anti-AQP4 antibodies results. One marked difference that sets MOG antibody-associated demyelination apart from both MS and AQP4 antibody-positive demyelination is the prognostic concerns, leading to the different amounts of aggressiveness in terms of management and treatment [7]. The target for anti-MOG antibodies are oligodendrocytes, which cause acute demyelinating lesions and are associated with good recovery potential. However, in NMOSD, anti-AQP4 antibodies target astrocytes, leading to lesions with a poorer prognosis for the patient. Other distinct characteristics of MOGAD are earlier age of onset, equal female-to-male ratio, and a thicker retinal nerve fiber layer [8-10]. 

In terms of imaging, practitioners should perform an MRI of the brain and orbit with contrast enhancement in all cases if available [11]. It is the gold standard to confirm the presence of ON, rule out any other intracranial pathology, and look for features of demyelination. Analysis of the blood and CSF excludes any signs of infection and inflammation. When suspecting demyelinating disease, practitioners should order serum testing for anti-AQP4 and anti-MOG. Another modality to investigate is optical coherence tomography (OCT) to look at the thickness of the retinal nerve fiber layer. Visual evoked potential (VEP) is also a useful tool in investigation; however, it is not performed in this case as it is not readily available. For a more systematic approach, Ramanathan et al. has proposed a comprehensive algorithm for investigation and diagnosis for the first episode of ON [8]. 

To our knowledge, there has been no clinical trial made on the treatment of ON in pediatric populations given its rarity of presentation. Much of the current practice in treating pediatric ON comes from understanding of the Optic Neuritis Treatment Trial study in adults [12-13]. The commonly described regime involves treatment with intravenous methylprednisolone 30 mg/kg/day for 3-5 days. This is followed by a course of oral prednisolone at 1 mg/kg/day, which will be slowly tapered over 4-6 weeks. Practitioners should monitor the side effect of steroids during these periods. An alternative treatment is using intravenous immunoglobulin and plasma exchange, for example, in steroid-resistant patients [12-15]. 

By now, we know that pediatric ON patients have good visual recovery despite having poor visual acuity at presentation [12-15]. The visual recovery begins during the initial 2-3 weeks of treatment and is complete by 4-6 months. However, the effects can last up to two years. In 70%-85% of cases, patients achieve final visual recovery of 6/12 or better.

## Conclusions

In conclusion, although pediatric ON is rare, it is a neuro-ophthalmic emergency, which almost always presents with profound initial visual loss compared to adults. Although in children it presents in such a grave manner, it is usually followed by encouragingly good visual recovery. This case report highlights the significance of investigating the secondary causes, emphasizing the need for a comprehensive examination and the diagnostic value of anti-MOG and anti-AQP4 antibodies.
